# Selective laser cleaning of microbeads using deep learning

**DOI:** 10.1038/s41598-025-99646-w

**Published:** 2025-04-30

**Authors:** Yuchen Liu, James A. Grant-Jacob, Yunhui Xie, Fedor Chernikov, Michalis N. Zervas, Ben Mills

**Affiliations:** Optoelectronics Research Centre, University of Southampton, Southampton, UK

**Keywords:** Femtosecond laser, Laser cleaning, Neural network, Real-time control, Laser material processing, Computational science

## Abstract

Laser cleaning is widely used industrially to remove surface contaminants with high precision. Conventional methods, however, lack real-time monitoring and feedback loops, often necessitating over-machining to ensure complete contaminant removal, which leads to inefficient energy use and potential substrate damage. In this work, we demonstrate a concept of selective laser cleaning via the application of femtosecond laser pulses and polystyrene microbeads with a diameter of 15 μm. These microbeads model challenging scenarios in high-precision optical work and delicate surface treatments across laboratory and production settings. To enable adaptive, real-time cleaning, we integrated a neural network that predicts the sample’s appearance after each laser pulse into a feedback loop, tailoring the cleaning process to a bespoke target pattern. This method ensures precise contaminant removal with minimal energy use, making it highly promising for applications demanding strict material control, such as wafer cleaning, sensitive surface treatments, and heritage restoration. By combining machine learning with ultrafast laser technology, our approach significantly enhances the efficiency and precision of cleaning processes.

**Supplementary Information:**

The online version contains supplementary material available at 10.1038/s41598-025-99646-w.

## Introduction

Lasers are routinely applied in various manufacturing processes, such as cutting^1^, welding^2^, drilling^3^and cleaning^4^, as they allow a precise and non-contact method of energy delivery. Although lasers efficiently deliver photons for manufacturing tasks, improving their energy efficiency remains economically and environmentally important. This is especially relevant in laser cleaning, where targets are often over-machined to ensure the complete removal of contaminants. This practice arises from the uncertainty regarding the type and thickness of contaminants, which may vary across the target and cannot always be determined in advance. Consequently, there is a clear demand for automated real-time control methods in laser cleaning to ensure that laser photons are utilized with precision and efficiency.

Laser cleaning technology provides the potential for a non-contact, precise, and environmentally friendly alternative to traditional methods like chemical cleaning, abrasive blasting and air jets with silica microspheres^5^. Since the invention of the first laser in the 1960 s^6^, research has concentrated on understanding the interactions between laser photons and materials^7,8^, forming the basis for the development of laser cleaning technologies. Among these interactions, photothermal and photochemical effects are particularly significant. These mechanisms involve the absorption of laser energy by contaminants or surface coatings, leading either to a rapid rise in temperature or to chemical reactions that transform the contaminant into a loose or more removable state. The resulting thermal energy can cause contaminants to vaporize or thermally decompose, while the loose state may facilitate removal with additional external assistance^9,10^. Early research in laser cleaning predominantly utilized nanosecond (ns) lasers and other long-pulse lasers, which effectively leveraged these interaction principles^11^.

In the 1990 s, laser cleaning began to emerge as a viable technology for cleaning of sensitive materials, with applications including the conservation of artifacts and historical objects^12,13^, and removing rust from steel^14^. These early systems demonstrated the effectiveness of nanosecond lasers in various contexts. However, they often faced limitations, such as reduced precision and an increased risk of damage to the underlying material, particularly for delicate substrates^15^. This was primarily due to the longer pulse durations, which could lead to localized heating and consequently undesired thermal effects. The subsequent development of ultrafast lasers, particularly femtosecond lasers, has brought significant advancements to the field. These lasers enable much greater precision, minimizing damage to the substrate while efficiently removing contaminants^16,17^. Their extremely short pulse durations significantly reduce thermal diffusion, making them ideal for applications involving thermally sensitive materials.

Laser cleaning technology has continued to evolve, benefiting from further refinement and integration with advanced systems such as automated and robotic platforms. Kostenko et al.^18^presented the design and validation of a remotely operated vehicle equipped with laser cleaning technology for efficient underwater inspection and biofouling removal from ship hulls, enabling maintenance without dry-docking. From the perspective of automation, deep learning is rapidly emerging as a transformative tool for advancing laser-based manufacturing^19^. Recent developments have highlighted its potential to accurately predict laser machining outcomes under diverse machining parameters. Mills et al.^20^ demonstrated an approach that enabled rapid identification of underlying material type, number of pulses and laser fluence by using a convolutional neural network (CNN) approach in real time. It also improves machining efficiency, provides predictive visualization of light-matter interactions and enables real-time monitoring and error correction in laser machining control systems. Grant-Jacob et al.^21^ presented a deep learning method for real-time monitoring of machining progress during femtosecond laser machining, accurately reconstructing surface conditions from plasma-generated images, allowing real-time process monitoring without direct sample observation. McDonnell et al.^22^ presented a neural-network-based approach for efficiently optimizing laser parameters and predicting surface texturing outcomes in short-pulse laser machining. Xie et al.^23^ studied that neural networks can monitor laser processing in real time by detecting beam translations and rotations with sub-pixel accuracy and triggering immediate feedback to halt machining. Behbahani et al.^24^ introduced an approach integrated with machine learning for predicting optimal picosecond laser parameters in alumina ceramic machining, effectively reducing experimental effort by accurately forecasting channel dimension from specific laser input condition.

In recent years, continued innovations in laser cleaning technology have been further propelled by breakthroughs in deep learning, leading to an expanding range of applications and enhanced process capabilities. Previous studies have demonstrated the use of machine learning for real-time monitoring of laser cleaning by analyzing acoustic waves^25^and flame^26^ characteristics to assess cleaning quality and surface roughness. Sun et al.^27^ showed that CNNs can effectively model the nonlinear laser cleaning process, predicting cleanliness from pre-cleaned images and laser parameters, while Hou et al.^28^ achieved comparable predictions of cleaning quality parameters using the support vector regression algorithm.

Although recent research has employed machine learning techniques for predicting post-laser surface conditions, monitoring surface roughness, and assessing cleanliness, the application of deep learning to achieve targeted irradiance and selective removal of individual contaminants in real time remains unexplored. In this work, we demonstrate the precise and efficient removal of microbeads enabled by deep learning, where a neural network is employed to predict laser cleaning outcomes within a real-time feedback loop. Microbeads possess uniform shape and size as well as stable optical properties, serve as an ideal model for simulating surface contaminants and optimizing removal processes. The use of microbeads ensures precise control and experimental repeatability^29^, which are essential for the reliability of this initial study. Potential contaminants in this context include microplastic (ranging from 10 to 50 μm), dust particles (typically around 10 μm in size), pollen grains (approximately 10 to 100 μm), and machining debris (often in ranging from 1 to 100 μm). These types of contaminants are commonly encountered in laboratory settings and production environments, where maintaining surface cleanliness is critical for ensuring the reliability and performance of advanced processes and applications. Addressing these challenges demands innovative solutions that balance precise cleaning control with minimal substrate damage, as highlighted in our comparison of laser cleaning methods (see Supplementary Table S1 online). This approach offers valuable insights into the optimization of target irradiance on the control substrate, underscoring the potential of deep learning to enhance the efficiency of laser cleaning and optimize photon usage.

## Methods

### Experimental setup

**Fig. 1:**
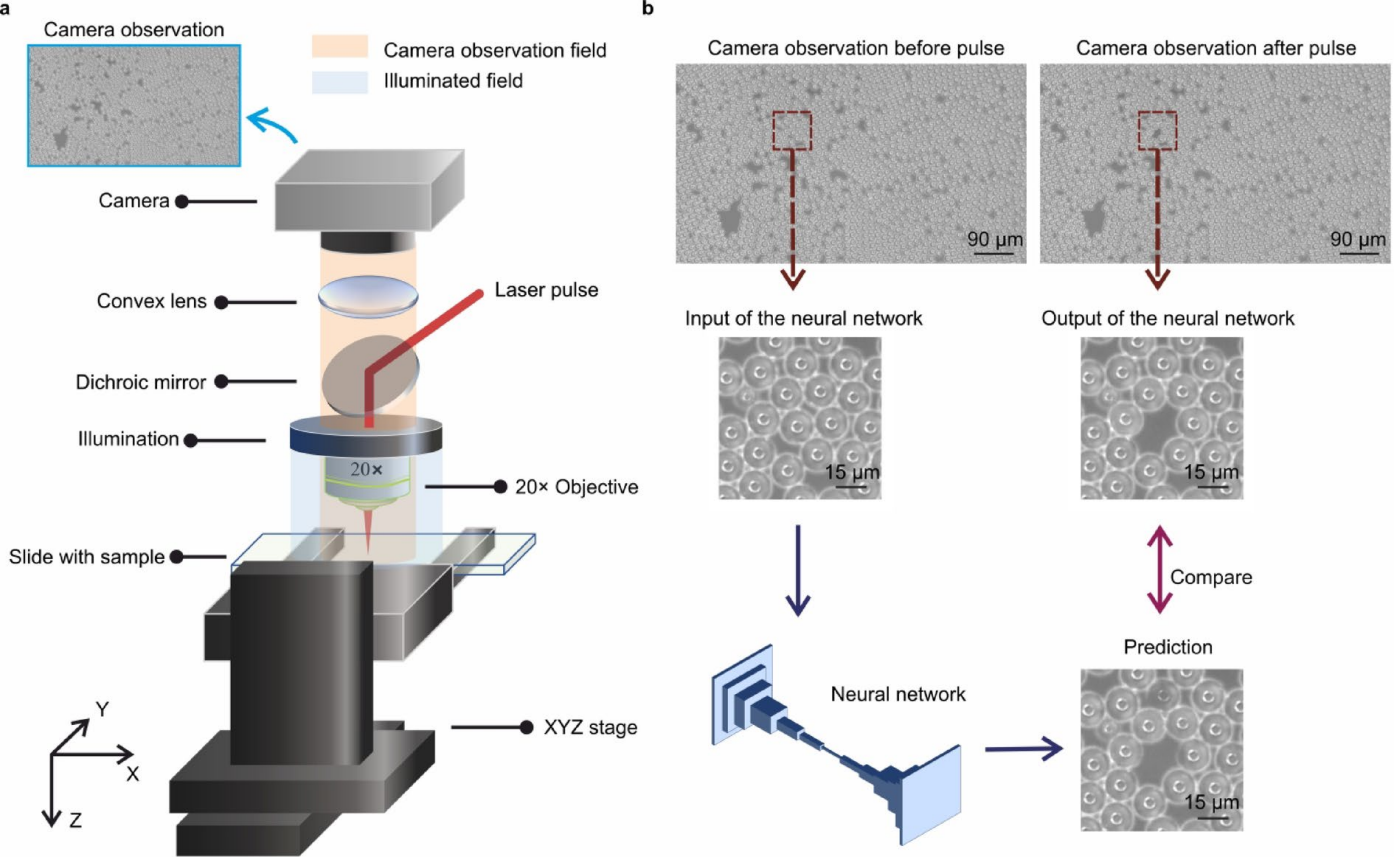
**a** The schematic of the experimental setup, and a camera observation of the sample without any laser incident where microbeads are aggregated. **b** A laser pulse removes a microbead (or microbeads) from the sample, and the neural network is trained to predict this cleaning outcome. The 3D image was created using Microsoft PowerPoint (Microsoft 365, version 2501, https://sotonac.sharepoint.com/teams/Office365).

Figure 1a presents the schematic of the experimental setup. Single pulses from a *Light Conversion Pharos SP* (190 fs, 6 W, 200 kHz, central wavelength 1030 nm) were directed onto the surface of a sample using a microscopic objective (*Nikon 20× MUE21200*). This sample consisted of a glass microscope slide (*Erie JMF*, *2950 WX-003*) coated with 15 μm diameter polystyrene (PS) microbeads (*Supelco*, *74964-5ML-F*^30^, which were used as simulated contaminants for selective laser cleaning. The microbeads were provided as a 10% aqueous suspension in water, with a density of 1.05 g/cm³. Samples were prepared by using a pipette pen to create 1 µL of PS solution. A glass microscope slide was chosen as the substrate primarily for its optical transparency, which supports clear evaluation of the sample’s condition before and after laser pulse exposure. The surface of the sample was monitored by a CMOS camera (*Basler*, *a2 A5320-23ucPRO*, 5320 × 3032, RGB) in real time. The sample was mounted on an XYZ motorized translation stage (*Zaber*, *LSM050 A-E03*) with a maximum travel distance of 5 cm on each axis, allowing precise movement of the sample relative to the laser focus. We determined through preliminary tests that a laser energy of 9 µJ, corresponding to a fluence of 2.17 J/cm² with a 23 μm spot size, effectively cleans individual microbeads without leaving the distribution unchanged, while also minimizing substrate damage and energy consumption. Single pulses were controlled via a pulse picker and Python software. Future work aims to adapt laser energy dynamically based on this foundation.

### Data collection

A dataset of training materials was generated using the experimental setup, which involved capturing paired images of the sample with a camera. The image pairs consisted of one taken before and another taken after the sample was exposed to a single laser pulse. After capturing each pair of observations, the translation stage moved the sample by 30 μm to a new position relative to the laser focus and camera. This data collection process was repeated 956 times, resulting in 956 pairs of camera observations. From each camera observation with a resolution of 5320 × 3032 pixels, a 512 × 512-pixel region of interest was cropped. This region was then applied with a 9 µJ laser pulse and resized to 256 × 256 pixels for further steps. These pairs of images were divided into two sets, referred to as the training set and a validation set, with a split ratio of 15 to 1. The network was trained on the training set with backpropagation enabled and was tested on the validation set with backpropagation disabled. The trained neural network was later employed in the experimental setup in real-time to control a laser cleaning process 16 days after training was completed. Figure 1b shows the camera observations of the sample captured before and after an incident laser pulse, used in training the neural network. The figure also includes the predicted cleaning outcome generated by the neural network, demonstrating its ability to accurately forecast laser cleaning results using only the camera observation prior to laser exposure were used as input data. The whole data collection (e.g., laser trigger, camera image capture and stage automation) and subsequent real-time laser cleaning process were controlled by a *Microsoft Windows 10* workstation, equipped with an *NVIDIA Titan Xp* (12 GB VRAM) GPU and an *Intel Core i7-7700* CPU @ 3.60 GHz.

### Neural network

The “pix2pix” architecture^31^, a conditional Generative Adversarial Network (cGAN), was employed to map camera observations of the sample before laser exposure to predicted observations after laser exposure. This architecture consists of two main components, the generator and the discriminator, which are both implemented as convolutional neural networks. The generator maps an input image (i.e., the camera observation of the sample before a laser incident) into a corresponding output image (i.e., the predicted camera observation of the sample after the laser incident), while the discriminator functions as a classifier, evaluating whether the generated image from generator is comparable to the actual training data (i.e., the ground-truth camera observation of the sample after the laser incident). This adversarial process enables the model to learn laser cleaning effects on 15 μm PS microbead distributions. We selected pix2pix for its proven efficacy in image-to-image translation, enabling us to predict post-laser pulse microbead distributions from pre-laser images with a balance of simplicity, modest data requirements, and adequate performance for this initial study. While architectures like SPADE^32^, SIMS^33^, and CRN^34^offer potential benefits such as improved visual quality or reduced model size, their documented complexity, greater data requirements, or slower inference times^35^render them less suitable for our current goals without task-specific optimization. For instance, SPADE and SIMS inference times exceed 100 ms for high-resolution outputs, and CRN’s cascaded approach scales similarly with resolution^35^. In this work, the pix2pix model was trained with a 7-depth (53 layers with input and output layers) of the generator and 4-depth (13 layers with input and output layers) of the discriminator, batch size of 2, a generator and discriminator learning learn rate of 0.0002 with an Adam (Adaptive Moment Estimation) optimizer^36^, for 100 epochs. The detailed overview of the network architectures used in this study is presented in the Supplementary Information (see Supplementary Table S2 and Table S3 online). The network was trained using MATLAB, and the inference time was estimated approximately 18.12 ± 9.96 ms on a *Microsoft Windows 10* workstation equipped with an *NVIDIA Quadro RTX 5000* (16 GB) GPU and an *Intel Core i9-9820X* CPU @ 3.30 GHz.

## Laser cleaning process

**Fig. 2:**
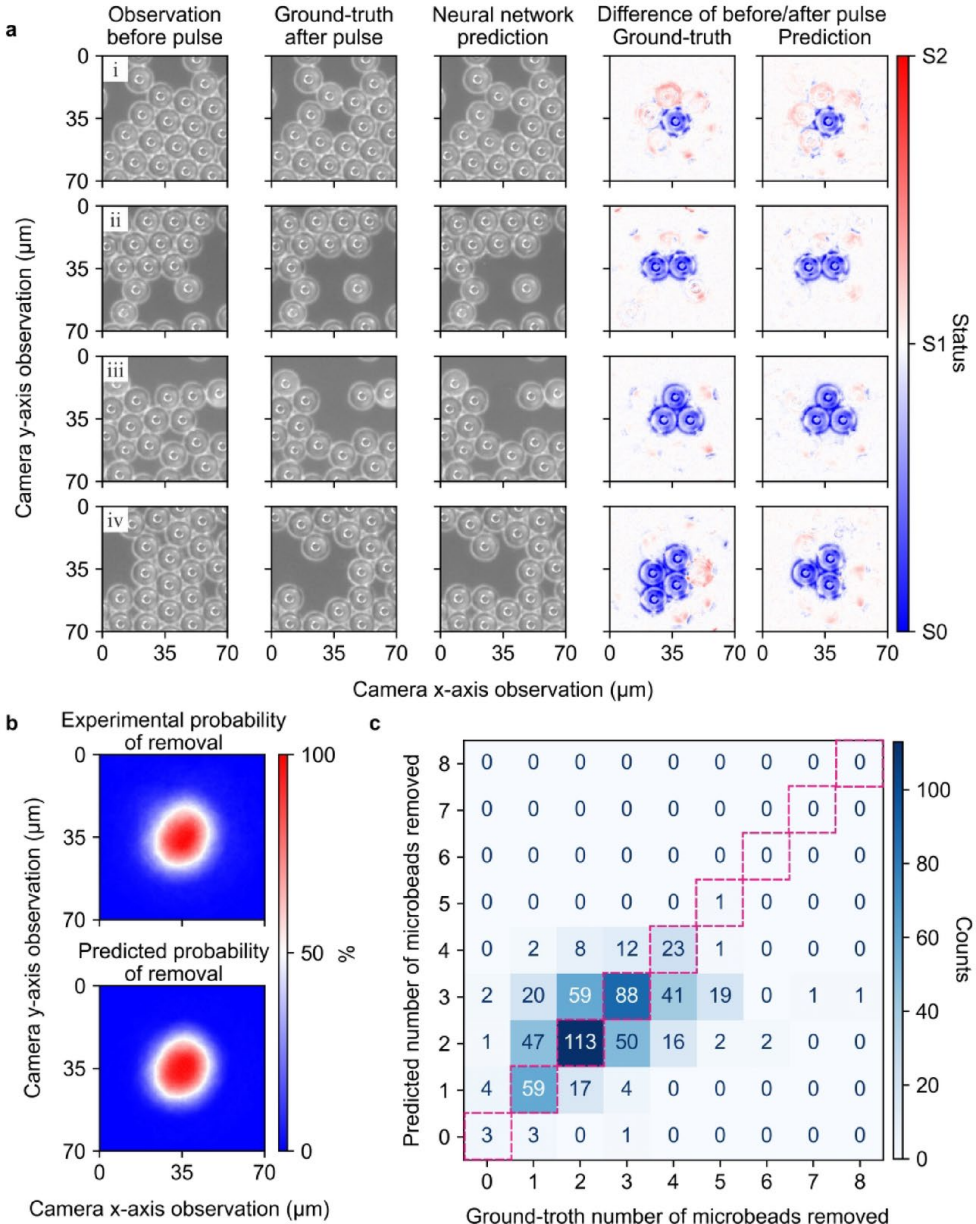
**a** (i-iv) Camera observations before and after an incident pulse that leads to the removals of 1, 2, 3, and 4 microbead(s) and their associated neural network predictions for each row. Blue/red map shows different behaviors of the microbeads: blue (microbeads are getting removed), red (microbeads have slight movement). **b** Estimated probabilities of microbead removals relative to the laser focus. **c** Confusion matrix showing the prediction accuracy of the network for the validation set.

Due to the complexity of light-matter interactions, a single laser pulse can remove multiple microbeads simultaneously. Figure 2a (i-iv) illustrates examples where 1, 2, 3, and 4 microbeads were removed by single laser pulses, respectively. Multiple factors could collectively contribute to the outcomes of microbeads removal, for instance, shockwaves and plasma induced by the light-matter interactions^4,9^, multiple reflections and refractions of the incident light between neighboring microbeads^37,38^, adhesion between contacting microbeads, and the spatial distribution of aggregated microbeads. It can be therefore argued that developing a practical analytical or semi-analytical model capable of deducing optimal actions for laser cleaning is exceedingly challenging. In this context, a data-driven method like a neural network provides a new approach by simplifying the laser cleaning control challenge into an image synthesis task that predicts the laser removal outcome.

Figure 2a, (i) to (iv) compares the predicted and ground-truth camera observations of the sample after laser pulses. The neural network is able to generate visually accurate predictions that closely resemble the ground-truth camera observations, preserving both the geometric characteristics of the microbeads and photographic properties such as color accuracy and white balance. The neural network demonstrates the ability to accurately predict key aspects of the laser cleaning process, including the number and spatial distribution of microbeads after each laser pulse. Interestingly, incident laser pulses not only remove microbeads but also cause slight displacements of surrounding microbeads. The neural network effectively captures these changes, as evidenced by pixel-wise difference comparisons between the camera observations taken before the pulse and the ground-truth/predicted camera observations taken after the pulse. However, in Fig. 2a (iv), an example is given where the neural network incorrectly predicts that three microbeads would be removed by the incident beam, whereas four microbeads were actually removed. This neural network was applied in the subsequent real-time work.

The neural network occasionally produces inaccurate predictions of laser cleaning outcomes, likely because it cannot access all necessary information, such as the adhesion between contacting microbeads and at the contact surface between microbeads and substrate. In other words, it is likely that hidden variables exist in the mapping between pre- and post-pulse observations, which commonly presented as probability. To prevent selective bias in data selection, we trained and tested 10 additional neural networks for analysis. In each training process, the training set and validation set was independently and randomly divided from the collected dataset. In this work, the presence of probability can be best visualized by examining the probability of microbead removal relative to its distance from the laser focus, as shown in Fig. 2b. The probability of removal here is estimated by the pixel-wise absolute pixel value differences between pre- and post-pulse camera observations. Although, as previously mentioned, the removal or displacement of microbeads can both lead to pixel changes, only the removed part was considered to make the probability graphs. This probability of removal estimation is performed on both the ground-truth and predicted camera observations. The results presented in Fig. 2b suggest, on both the real-word data and the generated data, that changes in pixels decrease as the relative distance between the position and the laser focus increases, which in turn could lead to the conclusion that a microbead is more likely to be removed if this microbead is closer to the laser focus (see Supplementary Figure S1 online and its description). Notably, the observed asymmetry in the probability distribution, likely due to optical aberrations in the beam delivery system, is also captured by the neural network.

The prediction accuracy for the neural network on the validation set is present in Fig. 2c in the form of a confusion matrix. On average, the neural network correctly predicted the outcomes of 47.8% of the test cases, 39.0% of the predicted cases had an error of one microbead (either over- or under-predicting), 11.3% of the predicted cases had an error of two microbeads, and 1.2% of the predicted cases had an error of three microbeads. To understand the reason for the accuracy of 47.8%, statistical analysis was conducted on the experimental images.

**Fig. 3:**
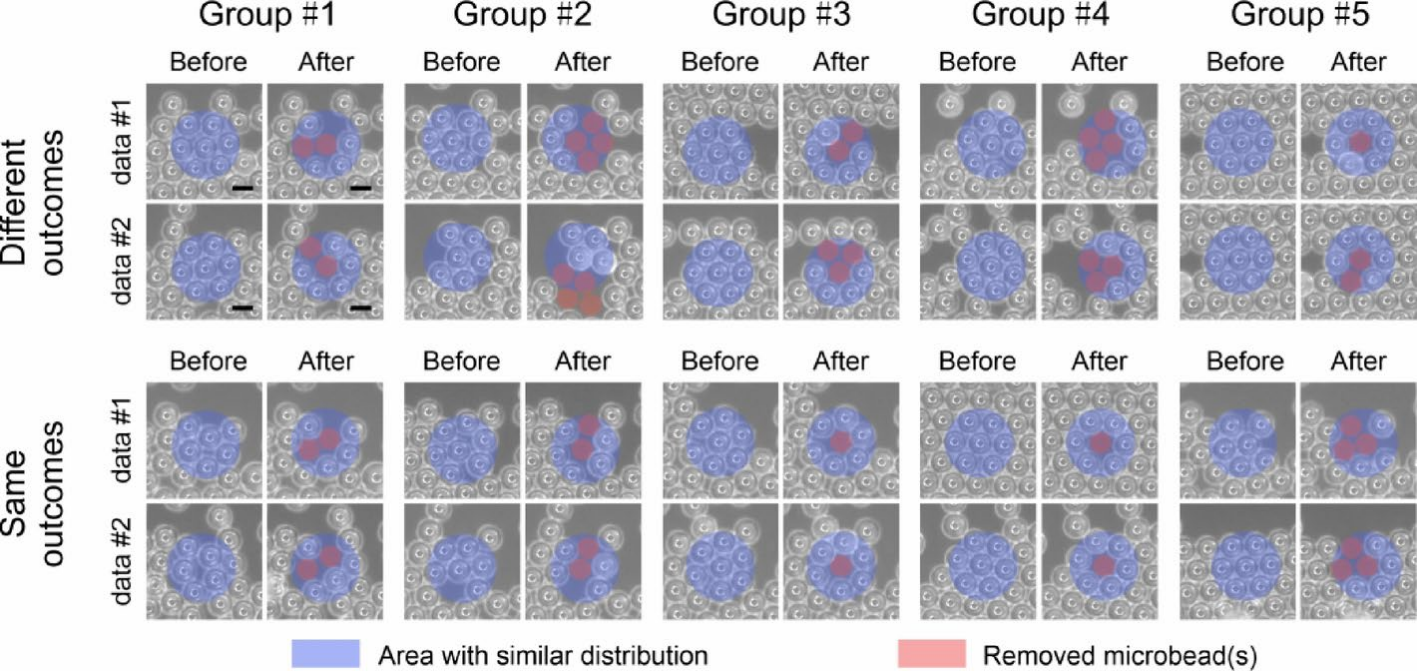
Experimental data for 15 μm PS microbead samples for before and after a 9 µJ femtosecond laser pulse, for cases where two examples of before images are similar but the associated after images are different (top row, “Different outcomes”), and cases where the before after images are similar (bottom row, “Same outcomes”). Five examples are provided for each case. The scale bar is 15 μm.

Figure 3 presents experimental data for 15 μm PS microbeads on a glass slide, captured before and after a 9 µJ femtosecond laser pulse, to illustrate the uncertainty in laser cleaning outcomes across five groups (Group #1 to Group #5). As identical microbead distributions are challenging to replicate experimentally, a mean squared error (MSE) metric was used to quantify the similarity of distributions in the experimental dataset. Pairs with similar distributions, identified via MSE, are marked with blue circles to indicate the target area. Removed microbeads are labeled with red circles. The figure includes two sets: the top row (Different outcomes) shows groups where similar initial distributions lead to different removal patterns, while the bottom row (Same outcomes) shows groups with similar initial distribution and similar cleaning outcomes. In analyzing prediction consistency, we found 59 sample pairs with similar microbead distributions: 33 pairs (56%) had matching cleaning outcomes, while 26 pairs (44%) differed. This variability suggests that hidden variables, which can not be observed directly from the image of the sample, may influence the laser cleaning process and hence limit the networks maximum prediction accuracy.

This variation underscores the intricacy of microscale laser interactions and impacts the predictive consistency of pix2pix neural networks. It has been demonstrated that transparent microbeads can function as lenses^37^, thereby scattering light in multiple directions. This phenomenon suggests the potential for interaction between the surrounding microbeads and the scattered light, though at a lower energy compared to the directly irradiated microbeads. The cleaning efficacy is contingent upon the relative position of the laser pulse with respect to the target microbead, under the assumption of a consistent laser pulse being applied to the same microbead. The figure illustrates the influence of laser interaction position on cleaning outcomes, thereby emphasizing the necessity of enhanced laser control and expanded training data to improve model accuracy under such dynamic conditions.

## Laser cleaning with real-time control loop

To demonstrate a practical implementation of this approach, the neural network was applied to real-time control of a laser cleaning experiment, 16 days after being trained. The objective was to show that the single neural network, trained to predict the appearance of the cropped sample after a single laser pulse, could also be used in an automated feedback loop to selectively remove microbeads from the sample in accordance with a bespoke target mask, and complete this task using the fewest number of laser pulses. This objective therefore demonstrates the capability of a data-driven automated technique for efficient laser cleaning, where the removal rate from each possible position on the sample can be predicted by the network at each step, and hence the optimal position of each subsequent laser pulse can be determined in real-time.

Importantly, as the neural network was trained to transform the camera observation before a pulse incident into a prediction that closely resemble the camera observations after the pulse incident, this prediction could then be used as a subsequent input to the same neural network, so that the cleaning effect of a second (or third or fourth etc.) pulse could be predicted (i.e., bootstrapping^39^. Therefore, as shown here, the neural network could also be used to simulate an entire laser cleaning experiment, with any number of laser pulses, if provided with an initial camera observation of the sample. As the network predictions in this work took 18 ms on average, this approach therefore also unlocks the potential for real-time modelling of laser cleaning.

**Fig. 4:**
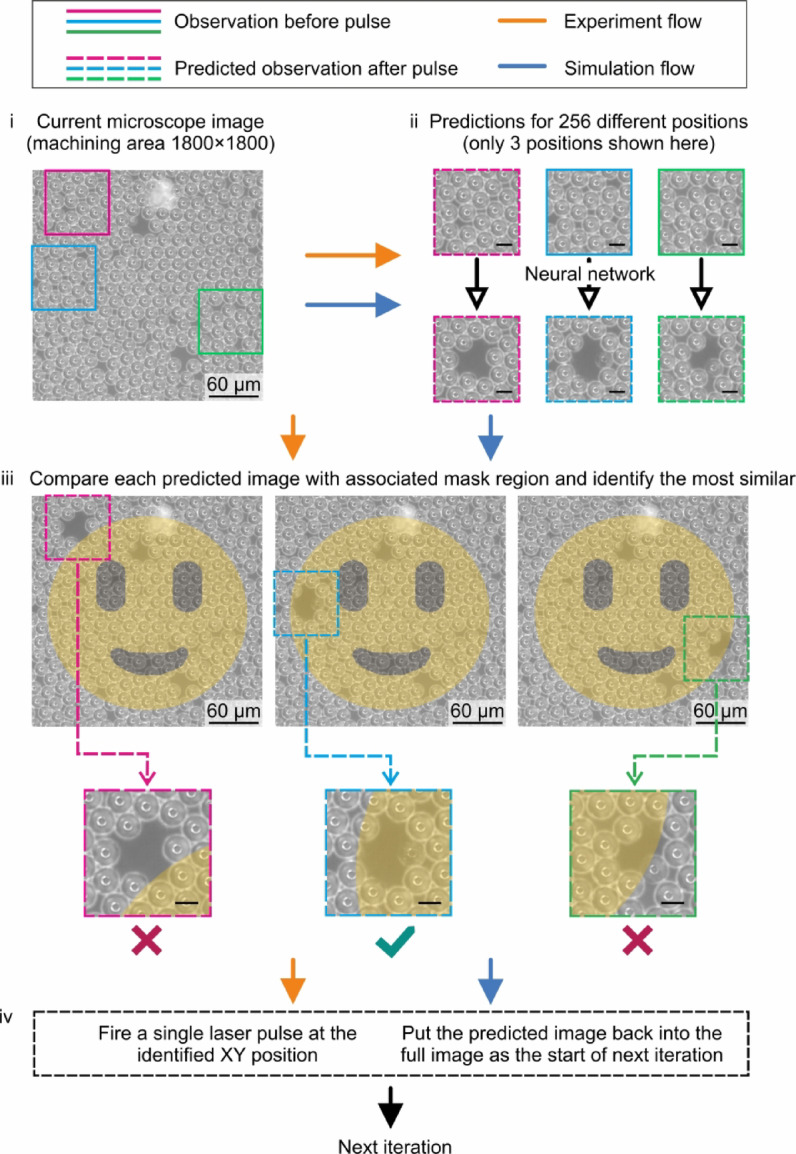
Flow chart showing the automated laser cleaning process for the experimental and simulated processes. (i) current machining area with magenta, blue and green squares that show three positions among the 256 positions; (ii) squares with solid line are the observation before a laser pulse, and with dashed line are the observation after a laser pulse; (iii) overview of the machining area with predicted images after laser pulse; comparing the predicted image with associated mask region to identify which position is optimal. Scale bar, 15 μm; (iv) two different actions before next iteration for experiment and simulation respectively.

Here, we use the nomenclature of experiment (where the network is used to predict the next optimal position, followed by a laser pulse, and the camera observation after the laser pulse is used as the sample state for the next iteration), and simulation (where the network is used to predict the next optimal position, and this prediction is used as the sample state for the next iteration). The two approaches are explained in Fig. 4. In both the experimental and simulation processes, the starting point was an initial camera observation of the sample, and the target mask was 1800 × 1800 pixels. Based on the previous method, we assume the laser targets the center of the cropped observation area, removing microbeads around this central region with each post-laser pulse prediction. Our goal is to ensure that each prediction enables the removal region to cover the entire pattern area. To achieve this, we cropped and resized the camera observation into 256 distinct 256 × 256-pixel images, each corresponding to a potential laser pulse position within a 16 × 16 grid, spaced 120 pixels apart, spanning a total area of 1800 × 1800 pixels (see Supplementary Figure S2 online and its description). Each of these cropped images was used separately as input to the neural network, to produce 256 predictions for the camera observation after a single pulse incident. A comparison was made between all 256 predictions and the 256 positions before the laser pulse. This involved directly applying pixel comparison and morphological image processing to isolate the removed microbeads (see Supplementary Figure S3 online and its description). The removed part was then compared pixel by pixel with the corresponding target mask, and the most strongly relevant position was chosen. The experimental process included an experimental laser pulse to clean the chosen position, while the simulated process included a predicted laser pulse on the chosen position. Due to inevitable aberrations in the beam delivery system, backlash-induced motion errors from the translation stages, erroneous predictions stemmed from the neural network, and cumulative errors from the bootstrapping processes, the sequence of laser pulse positions for the experimental and simulated processes therefore diverged as the laser cleaning process continued, despite starting from the same initial experimental microscope image.

**Fig. 5:**
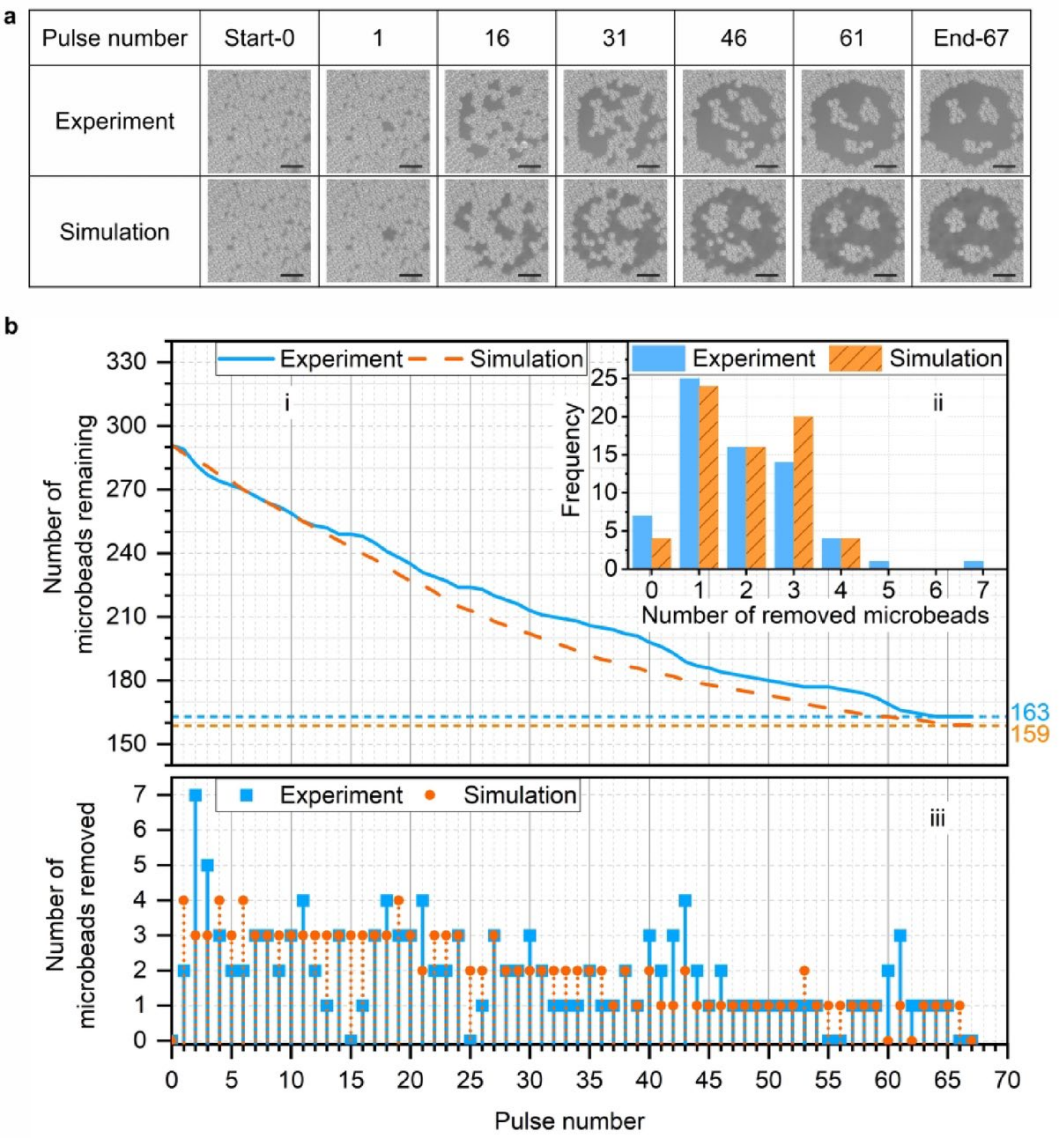
Real-time demonstration of experimental and simulated processes, when starting from the same initial experimental sample image. **a** The observation of cleaning area every 15 pulses. Scale bar, 60 μm. **b** (i) Number of remained microbeads with a subplot (ii); (ii) frequency of removing different number of microbeads; (iii) the number of microbeads removed in each pulse.

Figure 5 shows a comparison between a real-time demonstration of the experimental and simulation processes, when starting from the same initial camera observation and using a “smiley face” as the target mask. Figure 5a shows that while the experiment and simulation achieve a similar final result, they take different paths. Figure 5b (i-ii) show the removal rate and the frequency of removing a specific number of microbeads. In both cases, the process was considered to be finished when the number of microbeads and optimal position for the subsequent laser pulse were no longer changed. The results indicate that the simulated process has a relatively faster removal rate before 40 th pulse, which is attributed to the aforementioned differences in the experimental and simulation processes. There was a similar distribution of number of removed microbeads for the two approaches in Fig. 5b (ii), except for a slightly higher probability of zero microbeads, and five and more microbeads, removed experimentally. It is clear to observe that three microbeads are removed more frequently in simulation, since the network tends to predict the removal of three microbeads at the beginning of the cleaning. Figure 5b (iii) shows the absolute number of microbeads removed in each laser pulse during the two processes. The microbeads removed in each step has a relatively steady downwards trend for the simulation (from three microbeads to zero), and the experimental process had considerably more variation but did also follow this downwards trend. This downward trend is an important observation, as this demonstrates that the neural network predicts and prioritizes positions that correspond to the removal of as many microbeads as possible at each time step (but while still matching the target mask), and hence confirms that the neural network is directly enabling a more efficient laser cleaning process. In addition, the similarity between the final outcome and the number of laser pulses required for task completion provides strong evidence that the simulation process offers the potential for accurate real-time optimization of laser cleaning. In this work, 256 potential laser pulse positions were evaluated at each time step, and this could be increased if higher precision was needed in an industrial application.

**Fig. 6:**
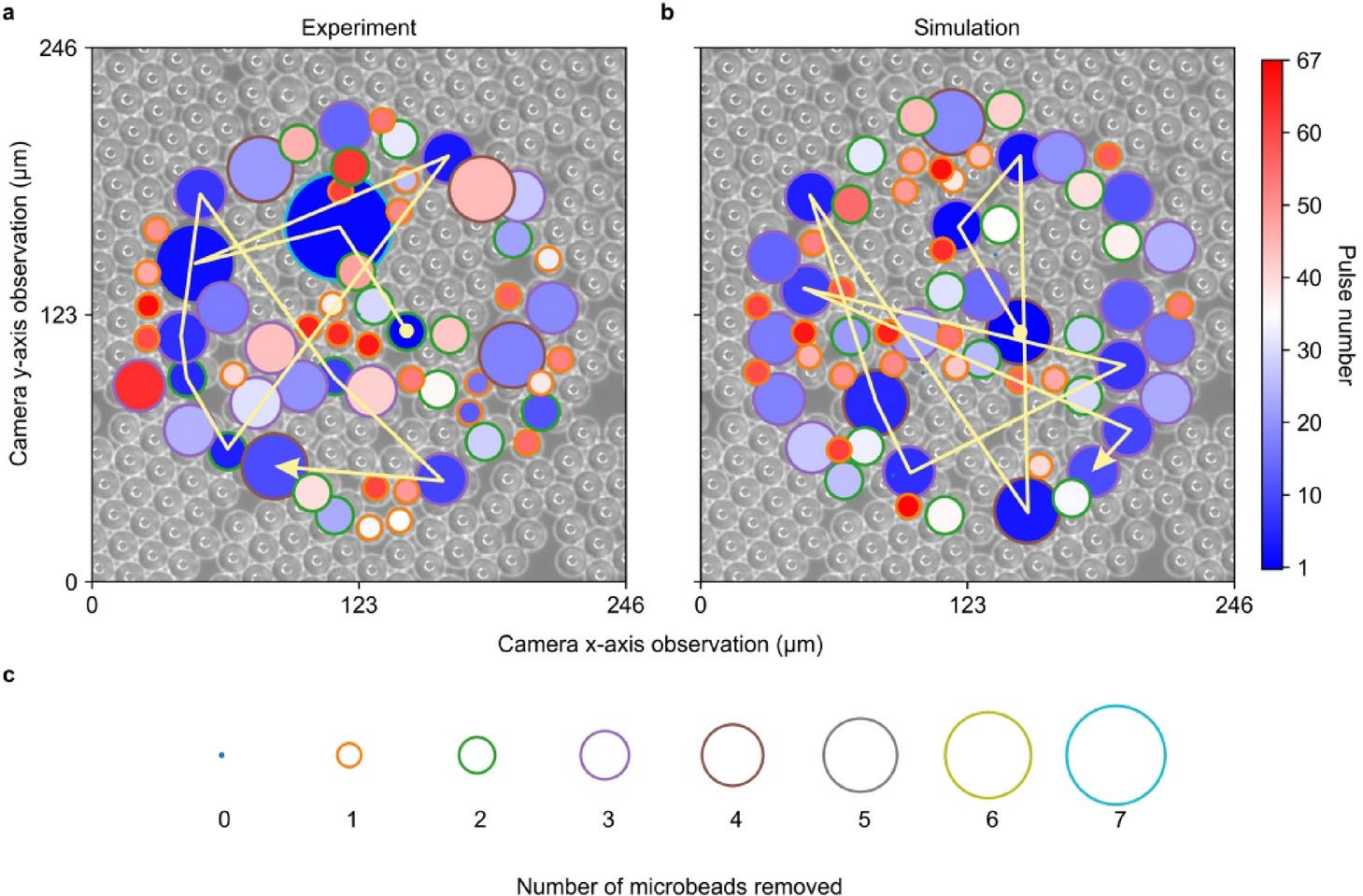
Spatial comparison of the experimental and simulated cleaning process. **a** Experiment cleaning process. **b** Simulated cleaning process. Color inside the circle indicate cleaning position in each pulse. **c** Circle contours in different size and color indicate different number of microbeads removed.

Expanding on the previous figure, which focused on data analysis of the cleaning process, Fig. 6a, b provides a more visually detailed comparison of the sequence of cleaning positions, in both experimental and simulation processes. The size of each circle shows the number of microbeads removed for that position, the color of each circle shows the pulse number, and the arrows highlight the path for the first ten laser pulses. The initial position for both cases is the same, as the initial sample is the experimental microscope image. However, the paths diverge after the second pulse, where the network predicts the removal of three microbeads, but seven microbeads are removed experimentally.

## Conclusion

In conclusion, we have demonstrated the real-time application of deep learning for automated, efficient laser cleaning of 15 μm PS microbeads. A pix2pix neural network was trained to predict post-laser pulse distributions after a 9 µJ femtosecond pulse, enabling selective microbead removal in a feedback loop guided by a bespoke mask pattern. This experimental setup, using microbeads as simulated contaminants, marks an initial step toward broader applications. Current results, with an inference time of 18 ms per sub-image, suitable for microscale lab cleaning but requiring optimization for industrial scales.

Future enhancements will increase versatility by varying contaminant size, material, and shape, aiming for selective cleaning that targets specific contaminants while preserving others (see Supplementary Figure S4 online and its description) under the different laser energies. This will involve refining laser energy control via deep learning, integrating topographical data into the model to enhance prediction accuracy, and exploring advanced architectures for contaminant detection or reinforcement learning for parameter optimization. Our current configuration, which includes an XYZ stage for translating the glass slide, a fixed femtosecond laser, and a camera aligned via an objective lens, is tailored for microscale cleaning tasks, in our case the removal of 15 μm PS microbeads. This design emphasizes precision, driven by the objective’s fixed focal plane, but sacrifices flexibility. To broaden its industrial applicability, we plan enhancements like real-time focus adjustment and galvo-scanner integration, enabling use on larger surfaces like aerospace components or expansive optical systems, thus combining precision with greater scalability. Potential industrial applications could include high-precision removal of dust in semiconductor fabrication clean rooms and the real-time cleaning of high-power laser optics, where this technique’s accuracy and energy efficiency could address critical contamination challenges. For industrial applications typically requiring sub-millisecond feedback, the deep learning model’s current inference time has room for improvement to optimize real-time performance. Future work will focus on optimizing the model by exploring strategies such as pruning the network to reduce complexity, distilling it into a smaller, more efficient version, pre-processing the data to enable a smaller network size, and reducing the field of view or resolution to lighten the computational load. Additionally, we plan to enhance performance through parallel processing to distribute tasks, leveraging dedicated hardware for acceleration, improving camera-computer connection speeds, and investigating dedicated FPGA systems for faster inference. These approaches aim to balance speed and accuracy for industrial-scale applications. These advancements will refine the technique’s potential for precise, energy-efficient surface decontamination across diverse conditions.

## Electronic supplementary material

Below is the link to the electronic supplementary material.

Supplementary Material 1

## Data Availability

Data underlying the results presented in this paper are available in Ref^40^.
