# Supplementary material for: Selective laser cleaning of microbeads using deep learning
Source: Sci Rep. 2025 Apr 30;15:15160. doi: 10.1038/s41598-025-99646-w (PMC12043982; doi:10.1038/s41598-025-99646-w)
Supplement: Supplementary file 1 — Supplementary Material 1 [file 41598_2025_99646_MOESM1_ESM.docx]

**Supplementary Material**

**Selective Laser Cleaning of Microbeads using Deep Learning**

Yuchen Liu*^1^, James A. Grant-Jacob^1^, Yunhui Xie^1^, Fedor Chernikov^1^, Michalis N. Zervas^1^, and Ben Mills^1^

^1^Optoelectronics Research Centre, University of Southampton, United Kingdom

* yl22u22@soton.ac.uk

# Comparative Analysis of Laser Cleaning Techniques

**Supplementary Table 1** Key features and relative trade-offs for three commonly used laser cleaning techniques, compared with the selective femtosecond laser cleaning integrated with deep learning technique presented in this work.

| Cleaning method | Continuous wave laser | Nanosecond pulsed laser | Laser-induced plasma/shockwave | Selective laser cleaning with deep learning |
| --- | --- | --- | --- | --- |
| Pulse duration | Continuous | Nanosecond | Nanosecond | Femtosecond |
| Precision | Low | Low | Moderate | Very high |
| Speed | Fast | Fast | Moderate | Slow |
| Cleaning scale | Large | Large | Moderate | Microscale |
| Energy efficiency | Low | Low | Moderate | High |

Supplementary Table S1 provides a qualitative comparison of the key features of three commonly used laser cleaning techniques, namely continuous wave laser, nanosecond pulsed laser, and laser-induced plasma/shockwave, with the novel selective laser cleaning with deep learning method presented in this work, focusing on pulse duration, precision, speed, cleaning scale, and energy efficiency. The continuous wave and nanosecond pulsed lasers, with low precision and low energy efficiency (estimated from typical power levels of 1-10 kW for continuous wave lasers ^1^ and fluence of 3.98 J/cm^2^ for nanosecond pulsed lasers ^2^), excel in speed and suit large-scale industrial applications like cleaning ship hulls. The laser-induced plasma/shockwave method, using nanosecond pulses, offers medium precision and energy efficiency, fitting medium-sized surfaces like artifacts. The proposed selective laser cleaning with deep learning, using femtosecond pulses, achieves high precision and energy efficiency (with femtosecond pulse energies of lower than 100 µJ in our case), but is slower and limited to microscale tasks like cleaning contaminants from optics, electronics and semiconductors.

# Architecture of the pix2pix^3^ model

**Supplementary Table S2** Generator Architecture ^4^. Detailed specifications of the generator network employed in the pix2pix experiments as described in the manuscript.

| Index of Layers | LayerName | LayerType | Description |
| --- | --- | --- | --- |
| 1 | inputImage | Image Input | 256×256×3 images |
| 2 | conv_down_1 | 2-D Convolution | 64 4×4×3 convolutions with stride [2 2] and padding ‘same’ |
| 3 | lrelu_down_1 | Leaky ReLU | Leaky ReLU with scale 0.2 |
| 4 | conv_down_2 | 2-D Convolution | 128 4×4×64 convolutions with stride [2 2] and padding ‘same’ |
| 5 | bn_down_2 | Batch Normalization | Batch normalization with 128 channels |
| 6 | lrelu_down_2 | Leaky ReLU | Leaky ReLU with scale 0.2 |
| 7 | conv_down_3 | 2-D Convolution | 256 4×4×128 convolutions with stride [2 2] and padding ‘same’ |
| 8 | bn_down_3 | Batch Normalization | Batch normalization with 256 channels |
| 9 | lrelu_down_3 | Leaky ReLU | Leaky ReLU with scale 0.2 |
| 10 | conv_down_4 | 2-D Convolution | 512 4×4×256 convolutions with stride [2 2] and padding ‘same’ |
| 11 | bn_down_4 | Batch Normalization | Batch normalization with 512 channels |
| 12 | lrelu_down_4 | Leaky ReLU | Leaky ReLU with scale 0.2 |
| 13 | conv_down_5 | 2-D Convolution | 512 4×4×512 convolutions with stride [2 2] and padding ‘same’ |
| 14 | bn_down_5 | Batch Normalization | Batch normalization with 512 channels |
| 15 | lrelu_down_5 | Leaky ReLU | Leaky ReLU with scale 0.2 |
| 16 | conv_down_6 | 2-D Convolution | 512 4×4×512 convolutions with stride [2 2] and padding ‘same’ |
| 17 | bn_down_6 | Batch Normalization | Batch normalization with 512 channels |
| 18 | lrelu_down_6 | Leaky ReLU | Leaky ReLU with scale 0.2 |
| 19 | conv_down_7 | 2-D Convolution | 512 4×4×512 convolutions with stride [2 2] and padding ‘same’ |
| 20 | bn_down_7 | Batch Normalization | Batch normalization with 512 channels |
| 21 | lrelu_down_7 | Leaky ReLU | Leaky ReLU with scale 0.2 |
| 22 | conv_up_7 | 2-D Transposed Convolution | 512 4×4×1024 transposed convolutions with stride [2 2] and cropping ‘same’ |
| 23 | bn_up_7 | Batch Normalization | Batch normalization with 512 channels |
| 24 | drop_up_7 | Dropout | 50% dropout |
| 25 | lrelu_up_7 | Leaky ReLU | Leaky ReLU with scale 0.2 |
| 26 | cat_up_7 | Depth concatenation | Depth concatenation of 2 inputs |
| 27 | conv_up_6 | 2-D Transposed Convolution | 512 4×4×1024 transposed convolutions with stride [2 2] and cropping ‘same’ |
| 28 | bn_up_6 | Batch Normalization | Batch normalization with 512 channels |
| 29 | drop_up_6 | Dropout | 50% dropout |
| 30 | lrelu_up_6 | Leaky ReLU | Leaky ReLU with scale 0.2 |
| 31 | cat_up_6 | Depth concatenation | Depth concatenation of 2 inputs |
| 32 | conv_up_5 | 2-D Transposed Convolution | 512 4×4×1024 transposed convolutions with stride [2 2] and cropping ‘same’ |
| 33 | bn_up_5 | Batch Normalization | Batch normalization with 512 channels |
| 34 | drop_up_5 | Dropout | 50% dropout |
| 35 | lrelu_up_5 | Leaky ReLU | Leaky ReLU with scale 0.2 |
| 36 | cat_up_5 | Depth concatenation | Depth concatenation of 2 inputs |
| 37 | conv_up_4 | 2-D Transposed Convolution | 512 4×4×1024 transposed convolutions with stride [2 2] and cropping ‘same’ |
| 38 | bn_up_4 | Batch Normalization | Batch normalization with 512 channels |
| 39 | lrelu_up_4 | Leaky ReLU | Leaky ReLU with scale 0.2 |
| 40 | cat_up_4 | Depth concatenation | Depth concatenation of 2 inputs |
| 41 | conv_up_3 | 2-D Transposed Convolution | 256 4×4×768 transposed convolutions with stride [2 2] and cropping ‘same’ |
| 42 | bn_up_3 | Batch Normalization | Batch normalization with 256 channels |
| 43 | lrelu_up_3 | Leaky ReLU | Leaky ReLU with scale 0.2 |
| 44 | cat_up_3 | Depth concatenation | Depth concatenation of 2 inputs |
| 45 | conv_up_2 | 2-D Transposed Convolution | 128 4×4×384 transposed convolutions with stride [2 2] and cropping ‘same’ |
| 46 | bn_up_2 | Batch Normalization | Batch normalization with 128 channels |
| 47 | lrelu_up_2 | Leaky ReLU | Leaky ReLU with scale 0.2 |
| 48 | cat_up_2 | Depth concatenation | Depth concatenation of 2 inputs |
| 49 | conv_up_1 | 2-D Transposed Convolution | 64 4×4×192 transposed convolutions with stride [2 2] and cropping ‘same’ |
| 50 | bn_up_1 | Batch Normalization | Batch normalization with 64 channels |
| 51 | lrelu_up_1 | Leaky ReLU | Leaky ReLU with scale 0.2 |
| 52 | cat_up_1 | Depth concatenation | Depth concatenation of 2 inputs |
| 53 | Output | 2-D Convolution | 3 1×1×67 convolutions with stride [1 1] and padding ‘same’ |

**Supplementary Table S3** Discriminator Architecture ^4^. Detailed specifications of the discriminator network employed in the pix2pix experiments as described in the manuscript.

| Index of Layers | LayerName | LayerType | Description |
| --- | --- | --- | --- |
| 1 | inputImage | Image Input | 256×256×6 images |
| 2 | conv_D_1 | 2-D Convolution | 64 4×4×6 convolutions with stride [2 2] and padding ‘same’ |
| 3 | lrelu_D_1 | Leaky ReLU | Leaky ReLU with scale 0.2 |
| 4 | conv_D_2 | 2-D Convolution | 128 4×4×64 convolutions with stride [2 2] and padding ‘same’ |
| 5 | bn_D_2 | Batch Normalization | Batch normalization with 128 channels |
| 6 | lrelu_D_2 | Leaky ReLU | Leaky ReLU with scale 0.2 |
| 7 | conv_D_3 | 2-D Convolution | 256 4×4×128 convolutions with stride [2 2] and padding ‘same’ |
| 8 | bn_D_3 | Batch Normalization | Batch normalization with 256 channels |
| 9 | lrelu_D_3 | Leaky ReLU | Leaky ReLU with scale 0.2 |
| 10 | conv_D_4 | 2-D Convolution | 512 4×4×256 convolutions with stride [2 2] and padding ‘same’ |
| 11 | bn_D_4 | Batch Normalization | Batch normalization with 512 channels |
| 12 | lrelu_D_4 | Leaky ReLU | Leaky ReLU with scale 0.2 |
| 13 | outputLayer | 2-D Convolution | 1 1×1×512 convolutions with stride [1 1] and padding ‘same’ |

# Real-time cleaning control


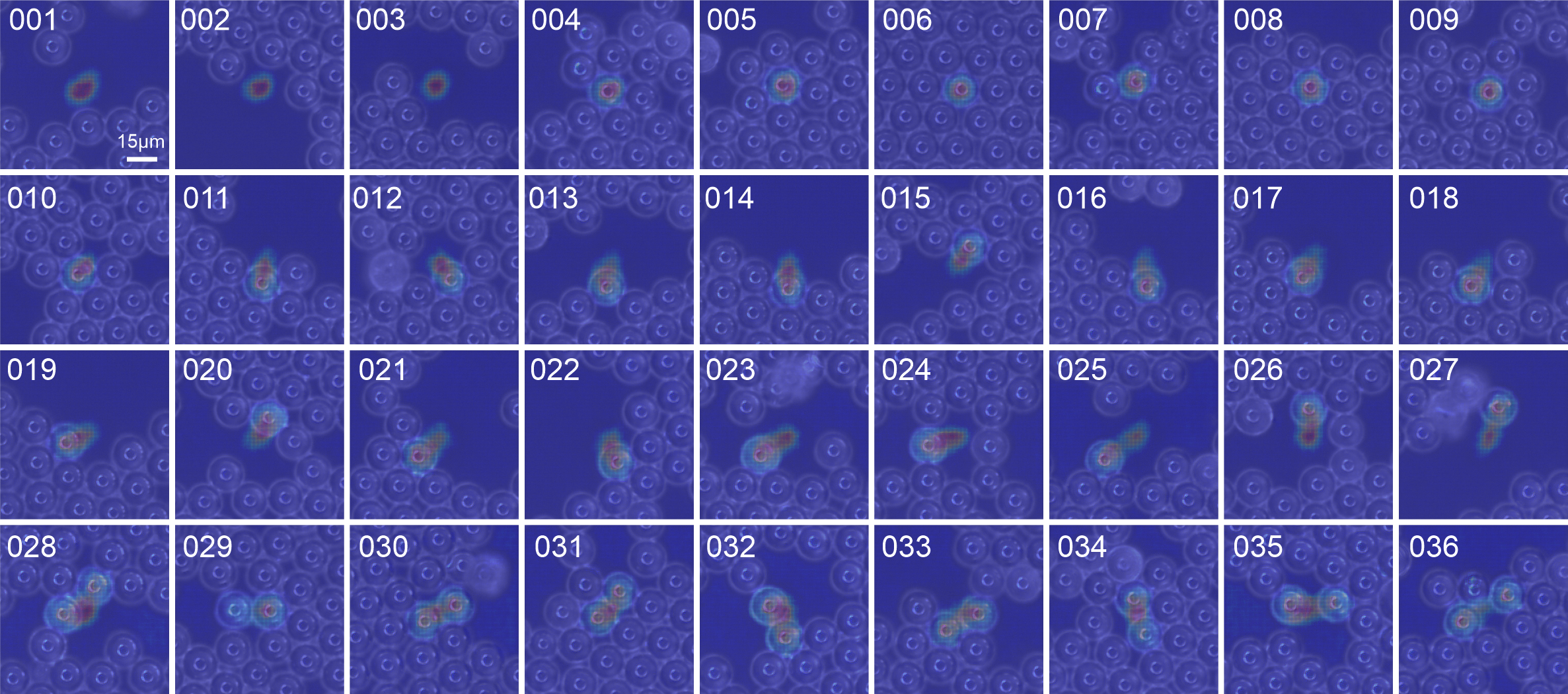


**Supplementary Figure S1** Thirty-six examples of experimental pre-laser pulse images overlaid with Grad-CAM calculations, which can be used to identify the regions of the pre-laser pulse images that are most important for neural network when making its prediction.

Supplementary Figure S1 demonstrates the application of Grad-CAM ^5^ (Gradient-weighted Class Activation Mapping) to identify the most influential regions in the neural network’s predictions for laser cleaning of 15 μm polystyrene microbeads on a glass slide after a 9 μJ femtosecond laser pulse. The Grad-CAM heatmaps highlight the approximate area of laser-matter interaction, indicating that the neural network heavily relies on this image region to predict the dynamics of the laser-microbead interaction and identify which particles will be removed. These findings shed light on the decision-making process of pix2pix, reinforcing its capability to model laser-induced effects for real-time cleaning applications.


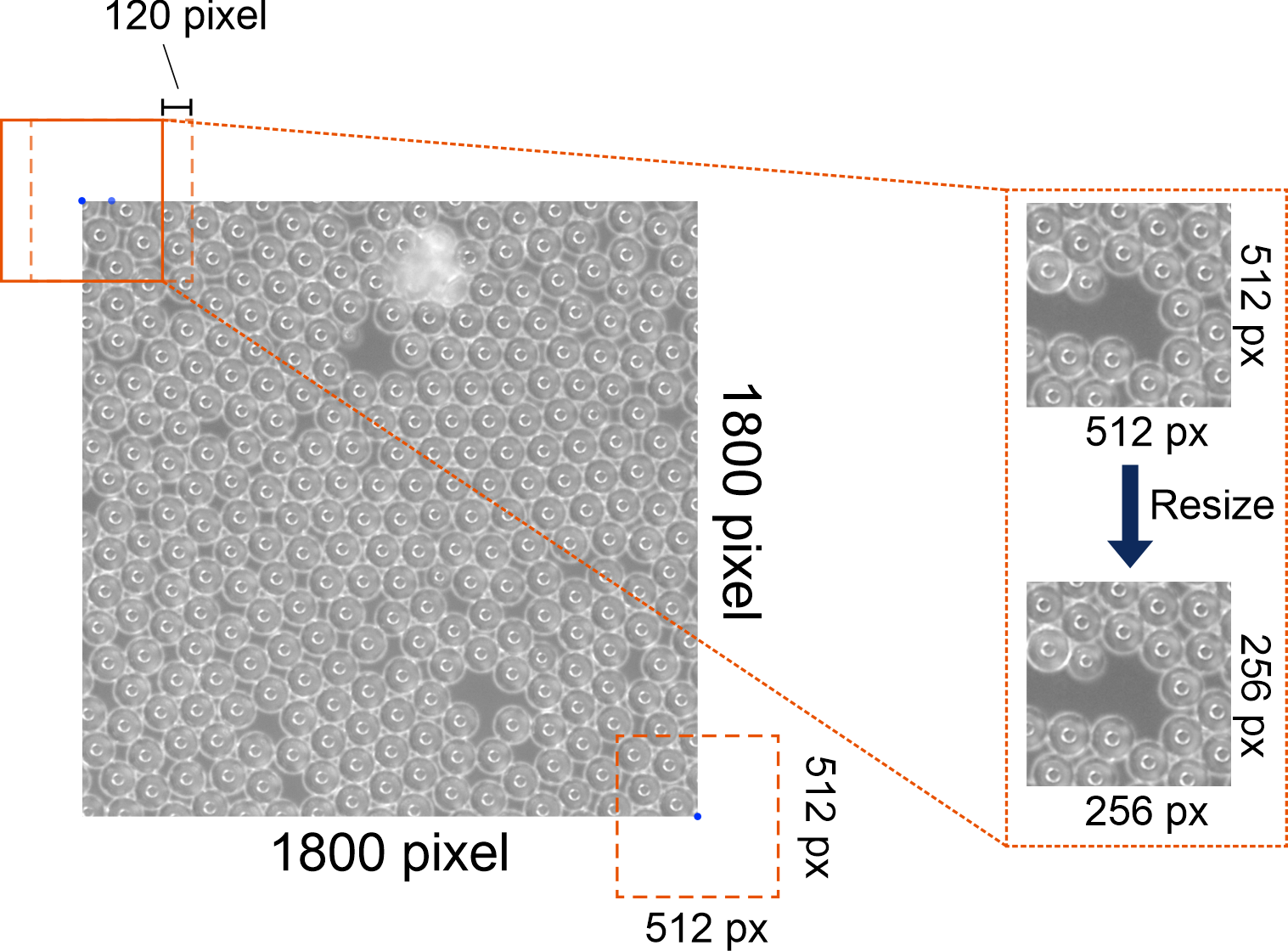


**Supplementary Figure S2** Method of cropping and resizing to make the input microscope images suitable for the neural network during the real-time feedback loop. The orange square indicates the cropping size (512×512 pixels) for each instance. The pattern area highlights the region activated for real-time cleaning.

Supplementary Figure S2 demonstrates the first step (Fig. 4 (i) in the manuscript) in real-time cleaning process in more detail. For real-time cleaning, an 1800×1800 pixels area is scanned, and 256 sub-images (512×512 pixels each) are collected per iteration in 120 pixels increments from top left to bottom right (as shown by the orange square). Then 256 sub-images are resized from 512×512 pixels into 256×256 pixels, matching the input requirement of the neural network. The neural network subsequently provides 256 prediction of post-laser pulse images, corresponding to each of the 256 input images, and hence corresponding to a prediction of the sample for laser pulses being incident at each of the 256 possible positions on the sample.

**
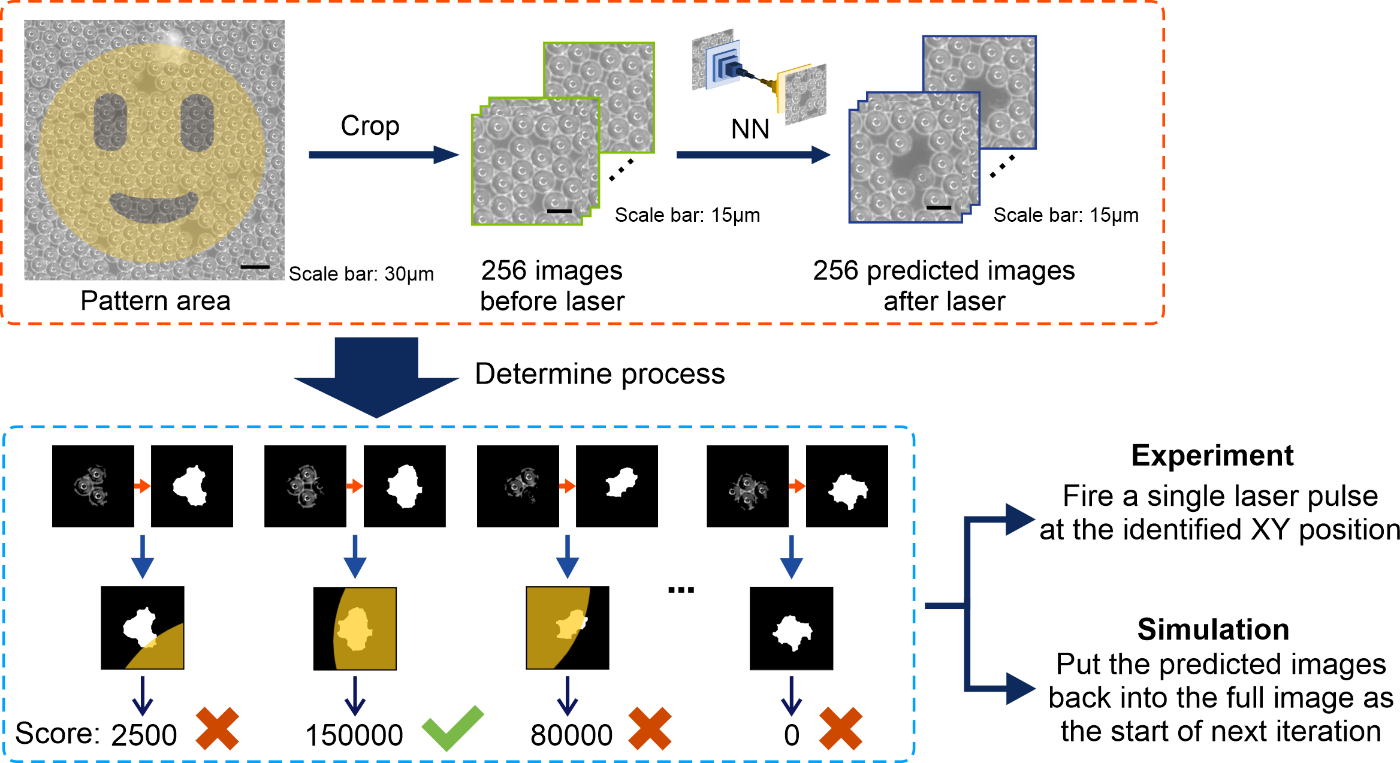
**

**Supplementary Figure S3** Overview of the mechanism for decision of the optimal position for the subsequent laser pulse, in the real-time control feedback loop. The section in the orange dashed-square is based on Fig. 3 in manuscript, and the diagram in the blue dashed-square demonstrates the image processing techniques applied during the decision making period to create a binary image that highlights the removed microbead(s).

Supplementary Figure S3 demonstrates the process of converting the removed region into a binary image, which can then be compared with the corresponding position of the mask. The dataset includes four distinct cases shown in Fig. S3: (farthest left) most of the component lies outside the region of interest; (left) the entire component is within the region of interest; (right) most of the component is within the region of interest; (farthest right) the entire component lies outside the region of interest. The score is determined by the size of the overlapping area, with a larger overlap corresponding to a higher score. After assessing all 256 positions, the position with the highest score is selected as the optimal target for the subsequent laser pulse.

In the simulation approach, which allows the neural network to predict the entire multi-iteration cleaning process, all steps occur computationally. Following the determined process, a ‘virtual’ laser pulse is simulated to remove microbeads, with the neural network prediction substituting for actual laser action. This simulation enables accuracy and stability testing, complementing the experimental workflow as a progress monitoring tool.

# Scalability of current configuration


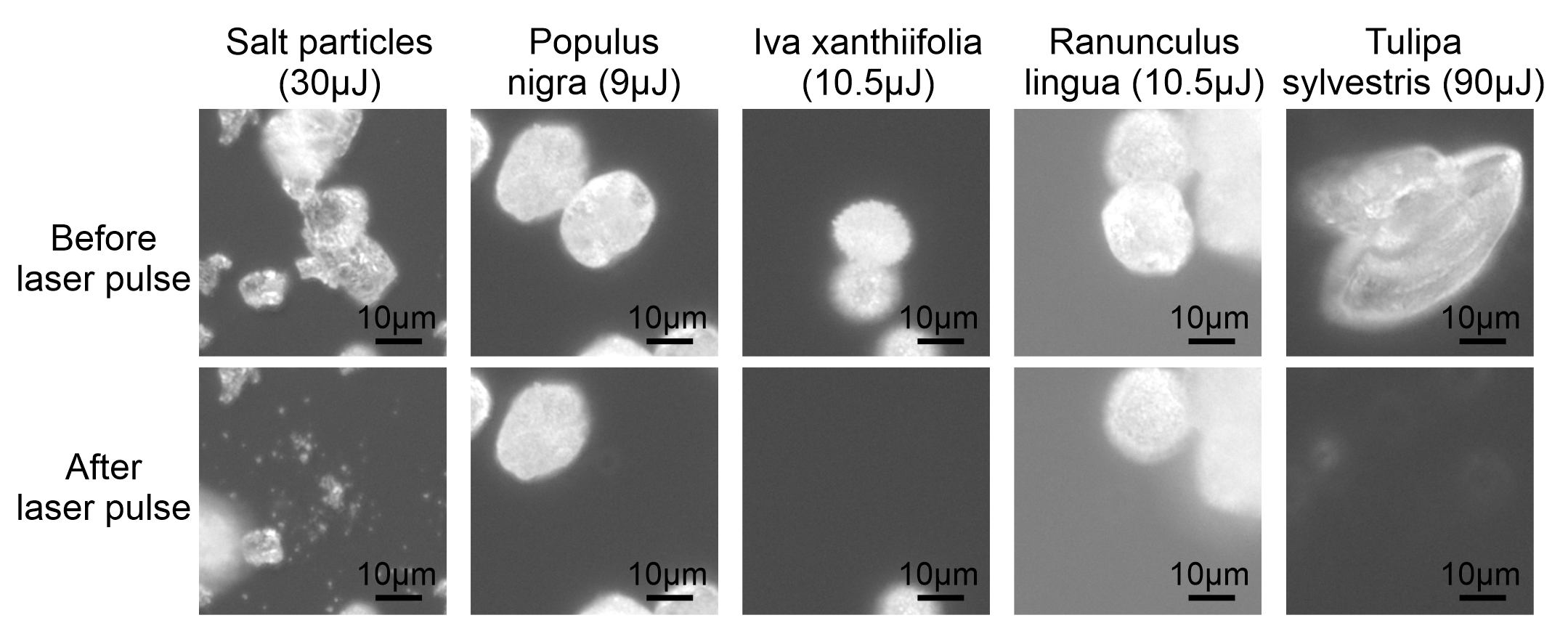


**Supplementary Figure S4** Images of diverse contaminant samples before and after a single femtosecond laser pulse at different laser energies, including salt (NaCl) particles, and Populus deltoides, Iva xanthiifolia, Ranunculus, and Tulipa pollen grains, demonstrating the potential for applying the demonstrated real-time cleaning process to a diverse set of contaminants.

Supplementary Figure S4 presents paired images of diverse contaminant samples on a glass slide, before and after exposure to single femtosecond laser pulse at different laser energies. The samples include salt (NaCl) particles, Populus deltoides, Iva xanthiifolia, Ranunculus, and Tulipa pollen grains, selected to showcase a range of particle types differing in composition, size, and morphology. Each image pair illustrates the effect of the laser pulse on the respective contaminant, with some particles removed or altered depending on their properties and position relative to the pulse. This diversity demonstrates the potential for the application of real-time selective femtosecond laser cleaning, beyond the initial demonstration of 15 μm polystyrene microbeads. These results support the future expansion of this technique to diverse contaminants, laying the groundwork for improved adaptability and precision in future cleaning applications.

# References

1 BaisonLaser. 10 Types of Laser Cleaning Methods You Need to Know - Baison. *url:* [*https://baisonlaser.com/blog/types-of-laser-cleaning-methods/*](https://baisonlaser.com/blog/types-of-laser-cleaning-methods/) (2023).

2 Li, Z. *et al.* Removal mechanism of surface cleaning on TA15 titanium alloy using nanosecond pulsed laser. *Optics & Laser Technology* **139**, 106998, doi:<https://doi.org/10.1016/j.optlastec.2021.106998> (2021).

3 Isola, P., Zhu, J.-Y., Zhou, T. & Efros, A. A. Image-to-Image Translation with Conditional Adversarial Networks. Preprint at <https://doi.org/10.48550/arXiv.1611.07004>. (2016).

4 Matlab_Deep_Learning. pix2pix. [*https://github.com/matlab-deep-learning/pix2pix*](https://github.com/matlab-deep-learning/pix2pix) (2020).

5 Selvaraju, R. R. *et al.* Grad-CAM: Visual Explanations from Deep Networks via Gradient-Based Localization. *2017 IEEE International Conference on Computer Vision (ICCV)*, 618-626, doi:<https://doi.org/10.1109/ICCV.2017.74> (2017).
